# Multimodal Imaging for the Detection of Brown Adipose Tissue Activation in Women: A Pilot Study Using NIRS and Infrared Thermography

**DOI:** 10.1155/2017/5986452

**Published:** 2017-09-14

**Authors:** Valentina Hartwig, Letizia Guiducci, Martina Marinelli, Laura Pistoia, Tommaso Minutoli Tegrimi, Giorgio Iervasi, Alfredo Quinones-Galvan, Antonio L'Abbate

**Affiliations:** ^1^Institute of Clinical Physiology, CNR, Via Moruzzi 1, 56124 Pisa, Italy; ^2^Fondazione G. Monasterio, CNR-Regione Toscana, Via G. Moruzzi 1, 56124 Pisa, Italy; ^3^Institute of Life Sciences, Scuola Superiore Sant'Anna, Piazza Martiri della Libertà 33, 56127 Pisa, Italy

## Abstract

**Purpose:**

A clear link between obesity and brown adipose tissue (BAT) dysfunction has been recently demonstrated. The purpose of this pilot study is to determine if near-infrared spectroscopy (NIRS) 2D imaging together with infrared thermography (IRT) is capable of identifying thermal and vascular response in the supraclavicular (SCV) areas after the ingestion of an oral glucose load as a thermogenic stimulation.

**Method:**

We studied two groups of women (obese versus lean) for discerning their different responses. NIRS and IRT images were acquired on the neck in the left SCV region during a 3 h oral glucose tolerance test (OGTT) and immediately after a cold stimulation.

**Results:**

We detected a significant thermal response of BAT in SCV fossa in both groups. Both during OGTT and after cold stimulation, skin temperature was persistently higher in lean versus obese. This response was not coupled with changes in oxygen saturation of subcutaneous tissue in that area.

**Discussion and Conclusion:**

The results show that NIRS/IRT may be a novel, noninvasive, radiation-free, easy to use, and low-cost method for monitoring, during the standard clinical practice, the diet and pharmacological intervention which aims to stimulate BAT as a potential therapeutic target against obesity and diabetes.

## 1. Introduction

Brown adipose tissue (BAT) is a key regulator in energy balance, protecting infants from hypothermia and a major contributor to diet-induced thermogenesis [1]. In newborns, BAT is mainly located around the neck and the interscapular regions. In the past, it was believed that BAT is lost after the first few years of life and so it has been considered unimportant in adults. Recently, with the emergence of metabolic imaging, it has been established that BAT persists beyond the neonatal period and is primarily located within the supraclavicular regions in adults [2–4].

Currently, the main methods available to assess BAT activity are positron-emission tomography (PET) and single photon emission computed tomography (SPECT) scanning. To also detect BAT presence, tissue biopsy can be used [5–7]. ^18^F-FDG-PET associated with computed tomography (PET-CT) is the current reference standard for the detection of activated BAT, but this technique is expensive and requires the administration of radiopharmaceuticals. Moreover, it can produce a large number of false positive results, as well as false negative results. Chemical shift MRI has been recently proposed as a noninvasive alternative to FDG-PET for the detection of BAT, but it does not allow for discrimination between active and inactive BAT [8, 9].

A clear link between obesity and BAT dysfunction has been recently demonstrated [10, 11]. However, the role of BAT in human metabolism and energy balance has not yet been fully understood. A practical and less expensive alternative to PET-CT to study BAT activation could be very useful. Since BAT is a thermogenic organ, recent studies have used thermal imaging [12] for detecting BAT activation [2–4].

Significant temperature changes in the supraclavicular (SCV) regions have been reported both in children and adults in response to cold exposure or meal ingestion. Moreover, Symonds et al. [13] demonstrated a highly localized increase in temperature within SCV following a standard cold challenge (by placement of the participant's hand in cold water) in healthy lean volunteers.

Recently, in a seventeen adult study, by using IRT, Jang et al. [14] found significant temperature changes in the SCV region and lateral upper chest after 2 hour cold exposure, those changes were related to PET-CT BAT-activation scanning.

In early 2016, van der Lans et al. [15] investigated the relation between supraclavicular skin temperatures and BAT activity values using a strictly temperature-controlled air-cooling protocol, founding a significant positive correlation between the changes in supraclavicular skin temperature with BAT activity.

Other studies used additional noninvasive techniques for the study of BAT, including near-infrared spectroscopy (NIRS) to obtain information about oxygen tissue consumption. Nirengi et al. [16] evaluated BAT density in adults using NIRS in the SCV region during 2 h cold exposure, but they found no changes in any NIRS parameters during the protocol. So, they compared NIRS parameters with mean standardized glucose uptake as assessed by ^18^F-FDG-PET/CT. They found a significant relationship PET parameter during cold exposure and NIRS-determined total hemoglobin concentration under thermoneutral conditions. Moreover, Muzik et al. [17] by using both 15O-PET and NIRS (for estimation of blood flow and oxygen consumption, respectively, in cold-activated human brown fat), found a significant relationship between PET-derived metabolic rate of oxygen uptake and NIRS in subjects with activated BAT.

Therefore, generally the thermogenesis and substrate disposal of the BAT have been investigated with cold exposure concluding that BAT is a potential therapeutic target against obesity and diabetes. The question, still controversial, to be further investigated is the evaluation of contribution of BAT to energy expenditure and substrate utilization when food activated defined “diet-induced thermogenesis (DIT)” to understand the role of BAT in conditions of thermoneutrality. Recently, Lee et al. [18] have found evidence linking human BAT activation to glucose-induced thermogenesis (GIT). GIT (also called thermic effect of glucose) is expressed as the increase in energy expenditure (measured by means of indirect calorimetry) which occurs after ingesting a glucose load and is well established in literature [19, 20].

The specific aim of this pilot study was to determine whether NIRS/IRT is capable of identifying thermal and vascular response in the SCV areas using an oral glucose load as a thermogenic stimulation and of discerning the different response between lean and overweight subjects. We performed a pilot BAT study on two small groups of lean and overweight adult women using both NIRS and IRT. Images by both techniques were acquired on the neck in the SCV region during a 3 h oral glucose tolerance test followed by a cold stimulation of the left hand. This work is part of a larger project that has the aim to investigate the role of BAT by noninvasive imaging methods in energy metabolism under thermoneutral conditions.

To our knowledge, this is the first study that uses the combination of NIRS with IRT for the imaging of BAT in adult humans.

## 2. Materials and Methods

### 2.1. Infrared Thermography

**Figure 1:**
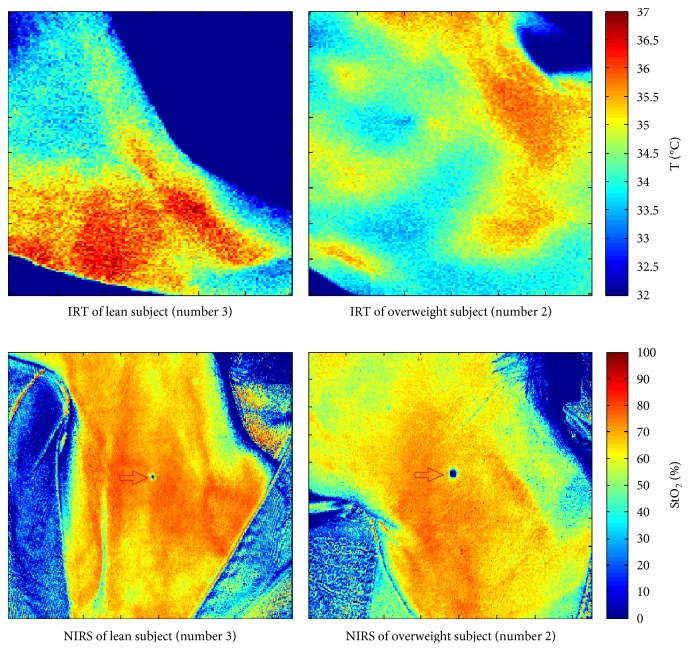
IRT and NIRS images for baseline condition (lean subject (number 3), overweight subject (number 2)).

Thermographic images of the neck in the left SCV fossa were acquired by using a Fluke Ti9Thermal Imagers (Fluke Corporation, Everett, WA). This technique is based on the principle that the amount of energy radiated depends on the surface temperature of the object and the emissivity of the object's surface [21]. The camera detects the infrared energy from an object and uses this information to estimate its temperature. The camera works well for surfaces that are efficient at radiating energy (high emissivity), such as the skin that has an emissivity factor of 0.98 [22]. The instrument used in this experiment has emissivity that is permanently fixed at 0.95, which works well for most surfaces but can provide very inaccurate reading if used directly on a shiny metal surface. Moreover, the infrared thermography camera has a 160 × 120 mm focal plane array; it operates in the 7.5 *μ*m to 14 *μ*m wavelength range, with 5% accuracy and a thermal sensitivity (NETD) ≤0.2°C at 30°C (200 mK). Thermographic images were acquired for each subject at different time steps (see Section 2.4); at each time step, the subject was positioned in the same position by means of references placed on the examination table and with a marker drawn directly on the area of interest (not shown in Figure 1). The marker was also used to choose the region of interest in the processing phase. In order to assure that the IRT camera was placed at the same distance from the subject every time, we used a reference on the area of interest to adjust the automatic focus of the camera.

### 2.2. NIRS

NIRS technique is based on two fundamental characteristics [23, 24]: relative transparency of human tissue to light in the near-infrared region (700–1000 nm) and the oxygenation-dependent absorption of oxyhemoglobin and oxymyoglobin (HbO_2_, MbO_2_) and deoxyhemoglobin and deoxymyoglobin (Hb, Mb). The NIRS signal from the skin is mainly derived from the hemoglobin contained in the blood flowing in the small vessels (arterioles, capillaries, and venules) present in the sampled area. Oxy- and deoxyhemoglobin measures permit calculating the overall oxygen saturation of hemoglobin (StO_2_) contained in the microvessels embedded in the explored tissue.

In the present work, a NIRS camera (Kent camera, Kent Imaging Inc., Calgary, Canada) is used to acquire two-dimensional maps of hemoglobin oxygen saturation in superficial tissues [25, 26]. The camera produces, in less than one second, the two-dimensional color-coded map of StO_2_ of the scanned surface and reports multispectral measurements for selected tissue regions. By simple touch-screen approach, an investigator can measure the value of StO_2_ in any selected region of the image immediately after acquisition. Then, postprocessing analysis of images allows comparison of oxygen saturation distribution at each time during the trial. The camera is mounted on a routable head (190 mm × 115 mm) at the extremity of the articulated arm: it has 470 LEDs with four wavelengths (670, 730, 890, and 940 nm) and a CCD receiver sensor. Each acquired image has a resolution of 750 × 480 pixels (162.5 × 104.0 mm). The images are exported in DICOM standard format for medical images. As stated by the manufacturer, the penetration depth of the NIR light is approximately 3 mm, so the camera permits to measure StO_2_ in subcutaneous tissues [27]. As already described for the IRT images, NIRS images were acquired for each subject at different time steps (see Section 2.4); at each time step, the subject was positioned in the same position by means of references placed on the examination table and with a marker drawn directly on the area of interest (see red arrows in Figure 1()). The marker was also used to choose the region of interest in the processing phase. In order to assure that the NIRS camera was placed at the same distance from the subject every time, we used the camera positioning indicators that help to set it at the proper height and location prior to taking a picture.

### 2.3. Subjects

Ten age- and sex-matched subjects were studied: five healthy women with low body mass index (age 35.2 ± 9.8, BMI = 19.6 ± 2.3 kg/m^2^) and five overweight healthy women (age 38.9 ± 10.3, BMI = 27.5 ± 1.8 kg/m^2^). The study was authorized by the local Ethical Committee. Each subject gave written informed consent.

### 2.4. Experimental Protocol

All subjects were selected from the Metabolic Unit of Fondazione G. Monasterio CNR-Regione Toscana, Pisa, Italy. Firstly, height and weight were measured and then BMI was calculated. Moreover, NIRS and thermographic baseline images were collected (after a 15 min acclimatization period).

A 3 h oral glucose tolerance test (OGTT) with the ingestion of 75 g of oral glucose [18] was performed for each subject. NIRS and IRT images were taken every hour during 180 minutes. At the end of OGTT, a cold stimulation was performed by immersing the subject's left hand in ice-water (5 to 9°C) for one minute [2, 28, 29]. Finally, NIRS and IRT images were acquired immediately after the cold stimulus. Each set of NIRS and IRT images was acquired with the subject in the same position in order to compare the area of interest StO_2_ and temperature values, respectively.

The ambient temperature was controlled and remained constant (20°C) for all measurements.

All the measurements were performed in the spring.

### 2.5. Image Processing

Both acquired NIRS and thermographic images are processed by using an IDL script specially designed. Oxygen saturation (%) and temperature (°C) values are extracted from a square (10 × 10 mm) region of interest (ROI) in the left SCV fossa that is known in literature as a BAT location in adults [2–4, 13, 30]. The ROI was chosen starting from the marker applied on the subject in the area of interest. The StO_2_ or temperature was calculated as mean of the pixels value included in the chosen ROI.

For each step of the experimental protocol (basal, 60′, 120′, and 180′ after glucose administration, immediately after cold stimulation) three consecutive NIRS/IRT images were acquired. At each time point in the measurement protocol, the mean of three consecutive images was reported as the value of oxygen saturation or temperature.

### 2.6. Statistical Analysis

Intragroup differences of StO_2_ and temperature over time were evaluated by Student's *t*-test for paired samples (variables were tested for normality using the Shapiro-Wilk normality test), while the differences in parameters between the two groups were evaluated by ANOVA.

A *p* value < 0.05 was considered to be significant. All analyses were conducted by using MATLAB.

## 3. Results

By way of example, Figure 1 shows the temperature image for baseline condition for lean subject number 3 and overweight subject number 2. Colour scale at the right side of the picture indicates the temperature values (°C). The corresponding NIRS image for the same subjects is also shown, where the colour scale at the right side of the picture indicates the oxygen saturation values (%). Both sets of images allow to clearly identify the left SCV fossa where choose the region of interest for the calculation of investigation parameters.

**Figure 2:**
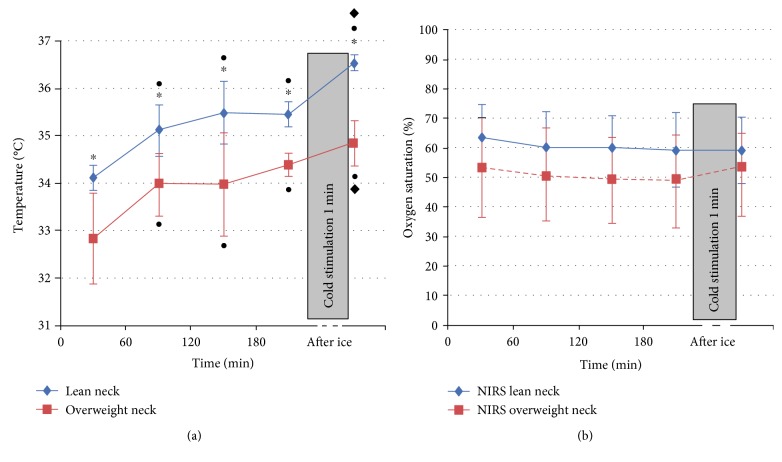
Temperature (a) and StO_2_ (b) values averaged on five lean subjects and on five overweight subjects, together with the 95% confidence intervals. The points on the graphs indicate the intragroup's significant differences of the measurements over the time versus the baseline value (*p* < 0.05), the diamonds indicate the intragroup's significant differences of the measurement after ice stimulus versus 180 minutes (*p* < 0.05), and the stars indicate significant differences between the two groups for the corresponding measurements at the same time step (*p* < 0.05). ^•^*p* < 0.05 for intragroup difference versus time 0; ^∗^*p* < 0.05 for difference between lean and overweight groups; and ^♦^*p* < 0.05 for intragroup difference versus time 180.

Figure 2 shows the temperature (Figure 2(a)) and StO_2_ values (Figure 2(b)) averaged on five lean subjects and on five overweight subjects (continuous blue line and dashed red line, resp.).

In both groups, a progressive increment of skin temperature was observed during OGTT. Relative to baseline, the positive thermographic response to glucose load was significant in the SCV region in both groups (Figure 2(a)). With respect to the value at 180 minutes after glucose ingestion, the SCV temperature after the ice stimulus is higher for both groups (*p* < 0.05).

Both during OGTT and after cold stimulation, skin temperature was persistently higher in lean versus obese (Figure 2(a)).

No changes were observed during both glucose load and after cold stimulation in the SCV fossa by NIRS imaging, and no differences were evident between the two groups.

## 4. Discussions and Conclusions

In this study, we designed a noninvasive multimodal imaging technique (NIRS and IRT) to identify and monitor thermogenesis related to human BAT in adults with different BMI. No previous studies have examined combined NIRS/IRT imaging as a tool for the determination of energy expenditure of BAT in adults. NIRS/IRT imaging has several advantages, first of all, the absence of radiation exposure, whereas PET-CT using is limited. The procedure is convenient for investigative studies in normal subjects.

We detected here, both in lean and overweight subjects, a consistent, and highly localized, increase in local temperature within the supraclavicular region induced by a glucose ingestion followed by a cold stimulus in thermoneutral conditions (20°C). Our findings confirm the results found by Lee et al. [18] in the same experimental conditions that provide evidence linking the human BAT activation to glucose-induced thermogenesis response during glucose challenge. We also found a significant difference in the SCV temperature of lean and overweight subjects with higher values in lean versus obese during OGTT. However, the SCV temperature values of the two groups are previously different in the baseline condition.

For our purpose, we considered the supraclavicular temperature value at 180 minutes after the glucose load as the baseline value for the evaluation of temperature increase due to the cold stimulus, since all the subjects are in the same condition at this time point. Interestingly, after cold stimulation, the two groups have a different response (*p* < 0.05).

Gatidis et al. [31] found a significantly higher local skin temperature of the supraclavicular region in individuals with active BAT with respect to temperature in individuals without active BAT. They hypothesized that probably the relatively increased supraclavicular skin temperature in lean individuals was caused by large blood vessels that are located in this anatomic area that conveys thermal energy from the body core to the extremities which might result in an improved detection in lean compared to obese individuals. However, in our study, the thermal response was not coupled with a change in oxygen saturation of the subcutaneous tissue in the same area. In fact, no significant differences in StO_2_ during OGTT and cold stimulation were observed in the SCV region of both lean and overweight subjects.

In Lee et al. [30], the authors used IRT to measure temperature overlying the SCV fossa after in response to cold exposure and meal challenge. They indicated as one major limitation of their study the uncertainty of whether changes in skin temperature overlying the SCV fossae arise from an increase in blood flow or from a thermogenic response in BAT. Our findings provide an answer to this uncertainty because NIRS images did not reveal any changes in tissue oxygenation. However, this could be due to the limitation of NIRS imaging in studying superficial BAT, particularly of the SCV fossa, but no differences in oxygen saturation response to stimulation occur even in lean subjects where the depth of subcutaneous fat is expected to be small. Moreover, the NIRS signal might be contaminated by contributions from other tissues, especially considering the highly heterogeneous nature of BAT.

In conclusion, NIRS/IRT may be a novel, noninvasive, radiation-free, easy to use, and low-cost method for monitoring, during the standard clinical practice, the diet and pharmacological intervention which aims to stimulate BAT as a potential therapeutic target against obesity and diabetes [32]. Further studies need to directly correlate changes in skin surface temperature with direct measurements of BAT activity and/or cutaneous blood flow and to verify if NIRS/IRT can provide real-time additional functional information with respect to information gained from static PET/CT images. Moreover, a number of technical challenges remain to be overcome in the use of NIRS/IRT to quantify BAT function, for example, the potential impact of skin depth over the supraclavicular region on skin surface temperature [33]. For example, local thickness of subcutaneous adipose tissue of the supraclavicular region could be measured by mean CT [31].
